# Abuse and mental disorders among women at walk-in clinics in Trinidad: A cross-sectional study

**DOI:** 10.1186/1471-2296-11-26

**Published:** 2010-03-30

**Authors:** Rohan G Maharaj, Colanne Alexander, Candace H Bridglal, Aysha Edwards, Hassina Mohammed, Teri-Ann Rampaul, Sharlene Sanchez, Gina P Tanwing, Kristy Thomas

**Affiliations:** 1 The Unit of Public Health and Primary Care, Faculty of Medical Sciences, The University of the West Indies, St. Augustine, Trinidad; 2 Faculty of Medical Sciences, The University of the West Indies, St. Augustine, Trinidad

## Abstract

**Background:**

To determine the prevalence of abuse by their partners and its association with mental disorders among female patients at walk-in clinics in Trinidad.

**Methods:**

Female participants from 16 randomly selected walk-in clinics, previously stratified to represent all administrative regions and urban and rural settings, who were 18 years or older, were surveyed during May to August 2007 using the WAST-Short and PRIME-MD questionnaires.

**Results:**

432 women participated (a 92% response rate), Of these 16% were aged 20-29, 11.8% 30-39 and 19% 40-49; 37% were married, 25% single; 44.7% were Indo- and 35% Afro-Trinidadian; 67.8% had achieved education up to age 14 only. 30.3% were employed and 3.0% reported incomes more than $5,001 TTD (Trinidad and Tobago Dollars) per month. Forty percent (173) of all respondents were positive for abuse as scored by the WAST-Short scale. Chi-square analysis suggested associations (*p *< 0.05) between abuse and age, employment status, being in a current relationship, and having the desire to cut down on alcohol intake. Further there were associations between abuse and the presence of depression, suicidal ideation, post-traumatic stress disorder and somatization as determined by the PRIME-MD scale. Logistic regression showed that the statistically significant (*p *< 0.05) predictors of woman abuse were age less than 49, wanting to cut down on alcohol use and currently being in a relationship.

**Conclusion:**

Among women of primarily lower socioeconomic status who attend walk-in clinics in Trinidad abuse as measured by the WAST-Short scale is high and there are statistically significant associations with mental disorders as determined by the PRIME-MD scale.

## Background

In Trinidad prevalence studies of domestic violence have been conducted in a variety of settings. Point prevalence rates in the general female population have been reported as16% [1,2], among currently pregnant, 9.2% [3], in the emergency room, 8% [4], and at the fee-for-service family practices, 27% [5]. Additionally, up to 40% of women at emergency rooms report a past experience of domestic violence. However we know little about the mental disorders or the long term consequences that may be associated with this abuse in Trinidad. Recently international studies have focused on the mental disorders associated with such abuse and reports suggest that there is a greater likelihood of depression [6], multiple somatic complaints [7] and post-traumatic stress disorder symptomatology [8].

Our objective was to determine the prevalence of woman abuse and the mental disorders associated with this experience, and further to explore which demographic factors may predict the likelihood of such abuse at walk-in clinics in Trinidad.

## Methods

In Trinidad there are seventy nine (79) health centres, most of which have walk-in clinics (where patients can present without an appointment for any medical problem). These clinics were stratified to represent all regional health authorities (administrative regions) and to capture rural and urban populations. Clinics were then selected using a table of random numbers; given the timeframe to conduct the interviews and the knowledge of attendance rates, 16 clinics were chosen according to the proportion of clinics per administrative region. Consecutive females attending these clinics were invited to participate if they were 18 years and older, willing to take part and had signed the consent form. Persons who presented with an emergency were excluded.

The WAST-Short (Woman Abuse Screening Tool-Short Form) was used to determine the presence of abuse among women. This tool has been shown to accurately detect 100% of non-abused women and 91.7% of abused women [9]. Only the 2 questions of the Short Form were used in an attempt control the number of questions in the survey instrument and to reduce respondent fatigue. The WAST-Short was used because a locally developed and validated scale was longer and had been found to have a low positive predictive value [5]. Additionally, the other major instrument in the questionnaire (see below) had a component for studying physical and sexual abuse.

The PRIME-MD PHQ (Primary Care Evaluation of Mental Disorders-Patient Health Questionnaire) has a specificity of 90%, a sensitivity of 75% and is used for the screening of minor psychiatric disorders and illnesses [10,11]. This questionnaire is based on the DSM-IV and has been used in many populations [12-15] and medical settings [16-19] and collects mental health, social and medical information and as noted above assisted in collecting information on physical and sexual abuse.

The WAST-Short scored as positive if a woman gave a response of *'a lot of tension'*, or *'some tension' *to the question: *'In general, how would you describe your present intimate relationship?' *Or if the woman completes the phrase: '*Do you and your partner work out arguments with*... with *'great difficulty' *or *'some difficulty'*. The combined questionnaire was piloted and found to be easily understood; inter-rater reliability was not tested. Responses to the PRIME-MD questionnaire were scored based on the "Reference Guideline for Interpreting the Patient Health Questionnaire and Brief PHQ" and the scores were used to determine the possibility of being at risk for having a mental disorder [20]. Demographic characteristics were also collected. Questionnaires were applied by trained interviewers. All authors except RGM carried out the interviews. Data were analysed using SPSS v.12. Associations between independent variables and the presence of woman abuse were analyzed using the chi-square test with a statistical significance of 5%. Regression analysis was carried out to determine which independent variables predicted the presence of woman abuse as defined by the WAST-Short.

This study was approved by the Ethics Committee of the Faculty of the Medical Sciences, The University of the West Indies (St. Augustine campus) and the relevant regional health authorities (RHAs) and was coordinated and supervised by the first author and conducted by medical students as part of their compulsory year-2 undergraduate research project. Patients were informed that the study was to understand common psychological and social problems among patients at the walk-in clinics and consent was obtained from all participants. Privacy was maintained by conducting the interviews on a one-to-one basis in a quiet part of the waiting room. However total seclusion was not possible because of the limited space and large volumes of patients at these clinics.

## Results

Four hundred and thirty two women responded (a 92% response rate), 49.5% were between 18 and 49 years; 37% were married. Indo-Trinidadians made up 44.7% and 35% were Afro-Trinidadian. Those who had achieved education up to age 14 only represented 67.8%, 30.3% were employed and 3.0% received more than TTD $5,001 per month ($1 USD = $6.3 TTD). Table 1 gives further details of the participants' demographic characteristics.

**Table 1:** Demographic description of 432 female participants in a survey of abuse and mental disorders in Trinidad.

Variable		N (%)
**Age Group (years)**	18-19	12 (2.8)

	20-29	69 (16.0)

	30-39	51 (11.8)

	40-49	82 (19.0)

	50-59	91 (21.1)

	60-69	87 (20.1)

	≥ 70	40 (9.3)

**Marital Status**	Married	160 (37.0)

	Single	108 (25.0)

	Divorced	21 (4.9)

	Separated	20 (4.6)

	Common law	57 (13.2)

	Visiting §	64 (14.8)

	Widowed	1 (0.2)

	Others	1 (0.2)

**Ethnicity**	Indo-Trinidadian*	193 (44.7)

	Afro-Trinidadian**	151 (35.0)

	Mixed	87 (20.1)

	Others	1 (0.2)

**Monthly Income**	<$1,000	100 (23.1)

	$1,001-$2,500	149 (34.5)

	$2,501-$5,000	52 (12.0)

	>/= $5001	13 (3.0)

	Does Not Know	85 (19.7)

	No Response	33 (7.6)

**Education**	Primary school (approximately Grade 5)	55 (12.7)

	School Leaving (Approximately Grades 5-6)	52 (12.0)

	Secondary School (approximately Grades 6 and above)	83 (19.2)

	Higher Education (Certificate/Diploma, Degree)	41 (9.5)

	Others	15 (3.5)

	None	186 (43.1)

**Employment**	Yes	131 (30.3)

	No	301 (69.7)

• * or ** Trinidadians of South Asian or African descent

• §A long-term relationship where a common home is not shared, participants may live at their family of origin or independent premises

Forty percent (173) of all respondents were positive for abuse as scored by the WAST-Short scale. Younger women were more likely to report abuse with those between the ages 20 - 49 reporting an average of 54.1% versus those between the ages 50 - 61 who reported an average of 31.4%. Women who were married (55.6%), in common-law relationships (40.7%) or single (66.7%) were more likely to report abuse versus 2% of all other groups. All major ethnic groups Indo- (37.8%), Afro-Trinidadians (44.4%) and mixed (37.9%) reported abuse. Higher proportions of women with lower educational achievements reported abuse (40.7%) versus 34.8% of women with higher achievements. However women with employment were more likely to report abuse (50.4%) versus 35.6% of unemployed. Similarly, among women who were in the higher income bracket, 53.9% reported abuse versus 39.6% of women in the lower income bracket. See Table 2.

**Table 2:** The association between demographic variables and the presence of a positive response on the Woman Abuse Screening Tool-Short form (WAST-Short).

	Positive response on WAST-Short	Positive response on WAST-Short	*p*-value*
	**Yes N (%)**	**No (%)**	

**Education**			**NS**
Lower achievement	157 (40.7)	229 (59.3)	
Higher achievement	16 (34.8)	30 (65.2)	

**Age**			**< 0.001**
49 years and less	115 (53.7)	99 (46.4)	
50 years and more	58 (26.6)	160 (72.4)	

**Employment**			**0.003**
Employed	66 (50.4)	65 (49.6)	
Un-employed	107 (35.6)	194 (64.4)	

**Income**			**NS**
< $4, 999 TTD	166 (39.6)	253 (60.4)	
$5, 000 TTD and more	7 (53.9)	6 (46.1)	

**Relationship**			**0.002**
Current (e.g. married, common-law)	127 (70.2)	154 (29.8)	
None (e.g. widowed, divorced)	46 (30.5)	105 (69.5)	

*Fisher's exact test

### Abuse and mental disorders

#### Violence and PTSD

Eight percent of all women reported physical violence being 'hit, slapped, kicked, or otherwise physical hurt or being forced into an unwanted sexual act' in the past 12 months. Additionally, 10.4% of all women reported symptoms suggestive of post-traumatic stress disorder (PTSD), being 'bothered a lot' and 15.2% 'bothered a little' by 'thinking or dreaming about something terrible that happened to them in the past'. There was a statistically significant association between reports of abuse and PTSD with 48.9% of the women who were 'bothered a lot' and 58.5% of the women who were bothered a little' reporting abuse, compared with 35.1% of respondents who were 'not bothered'.

#### Alcohol abuse

There was a statistically significant association between reports of abuse and women's report that they would like to 'cut down on their alcohol intake'. Sixteen (64%) of the 25 women having this wish reported that they had experienced abuse in the past year.

#### Depression

There was a statistically significant association between reports of abuse and both suicidal thinking and the scoring of major depression. See Table 3.

**Table 3:** The association between mental health symptoms and the presence of a positive response on the Woman Abuse Screening Tool-Short form (WAST-Short).

	Positive response on WAST-Short	Positive response on WAST-Short	*p*-value*
	**Yes** **N (%)**	**No** **N (%)**	

**Would like to cut down on alcohol use**			**0.011**
Yes	16 (64.0)	9 (36.0)	
No	157 (38.6)	250 (61.4)	

**Possible depression**			0.001**
Major depression	23 (69.7)	10 (30.3)	
No depression	117 (31.3)	206 (68.7)	
Other depressive disorder	33 (44.0)	42 (58.0)	

**Suicidal ideation**			**0.018**
Yes	12 (66.7)	6 (33.3)	
No	161 (38.9)	253 (61.1)	

**Possible somatization**			**0.014**
Yes	59 (48.8)	62 (51.2)	
No	114 (36.7)	197 (63.3)	

**Possible anxiety disorder**			**0.072**
Yes	14 (56.0)	11 (44.0)	
No	159 (39.1)	248 (60.9)	

**Possible Post Traumatic Stress Disorder**			0.001**
Not Bothered***	113 (35.1)	209 (64.9)	
Bothered a little	38 (58.5)	27 (41.5)	
Bothered a lot	22 (48.9)	23 (51.1)	

**Reported physical or sexual abuse in the past year**			**< 0.001**
	25 (71.4)	10 (28.6)	
Yes	148 (37.3)	249 (62.7)	
No			

*Fisher's exact test

** Pearson Chi-square

*** About something terrible that has happened in the past.

#### Somatization

There was a statistically significant association between reports of abuse among women with multiple somatic complaints when compared to those who did not have multiple somatic complaints, with 48.8% versus 36.7% respectively reporting abuse. Further analysis was carried out to describe which somatic complaints were more common among women who reported abuse, it was found that women who complained of being 'bothered a lot' in the past 4 weeks with either menstrual cramps, pain or problem with sexual intercourse, headaches, feeling the heart pounding or racing, shortness of breath or nausea, gas and indigestion were more likely to report abuse.

#### Anxiety and Panic

There was a statistically significant association between reports of abuse and anxiety where of the 25 women (5.8%) reporting symptoms suggestive of anxiety 56% reported abuse versus the 39.7% who reported abuse but had no anxiety symptoms. Of the 21 women with panic symptoms 52.4% had abuse versus 39.4% with no panic disorder. This was not statistically significant.

#### Additional analysis

Chi-square analysis suggested association (*p *< 0.05) between history of abuse and age, employment status, current relationship, the desire to cut down on alcohol intake, and the presence of symptoms of depression, PTSD and somatization. Tables 2 and 3 provide more details of the chi-square analysis. Logistic regression showed that statistically significant (*p *< 0.05) predictors of woman abuse were age less than 50, wanting to cut down on alcohol use and being in a relationship. See Table 4.

**Table 4:** Results of Binary Logistic Regression analysis to determine the independent variables associated with the presence of a positive response to the Woman Abuse Screening Tool-Short form (WAST-Short) among female attendees at walk-in clinics in Trinidad.

Independent Variable	Odds Ratio (95% CI)	*p *value
**Education**		
Lower *vs. *Higher achievement	1.57 (0.75-3.26)	NS

**Age**		< 0.001
>/= 50 *vs*. </= 49 y	3.42 (2.18-5.34)	

**Employment**		NS
Unemployed *vs*. Employed	1.48 (0.91-2.39)	

**Income**		NS
> $5000 *vs*. <$4 999 TTD/mo	0.69 (0.195-2.46)	

**Presently in a relationship**		< 0.001
No *vs. *Yes	2.63 (1.62-4.25)	

**Would like to cut down on alcohol**		0.045
No *vs. *Yes	2.48 (1.02-6.02)	

## Discussion

This study shows that abuse was common and was reported by 40% of female respondents at walk-in clinics in Trinidad and further 8% of all participants reported physical and sexual abuse in the last year. There were associations between abuse and age, employment status, current relationship, and the desire to cut down on alcohol intake. Further analysis suggested that at these primary care walk-in clinics, statistically significant predictors of woman abuse were being of an age less than 50, wanting to cut down on alcohol use and being in a relationship.

This paper supports previous studies that show that abuse is as common in this population as in primary care populations worldwide. Its results add to the Caribbean literature by exploring the association between abuse experienced by women in their relationships and mental disorders in the primary care setting. It further shows that among women who report abuse, depression, somatization and PTSD are common. Suicidal ideation also occurs more commonly among the women who report abuse than those who do not.

These findings are consistent with the recent medical literature [6-8,21].

Another potentially important finding is the relationship between the symptoms which 'bothered the participants 'a lot' during the past 4 weeks and the reporting of abuse, these symptoms included menstrual cramps, pain or problem with sexual intercourse, headaches, feeling the heart pounding or racing, shortness of breath or nausea, gas and indigestion. These are all common primary care symptoms. The lesson to take away is that if these symptoms are present and somatisation is considered then the physician should explore the possibility of abuse.

This paper has several strengths including the large sample of participants, the good response rate, and the random selection of clinics to represent the various administrative centres and urban and rural centres. The good response rate was thought to be due to 3 factors, the long waiting times at the clinics before patients saw the physician which meant that patients were not rushed and could easily complete the questionnaire before their consultation, the interviewers were all females and so participants may have been more comfortable talking about sensitive topics, and these clinics have been poorly studied in the local medical literature.

The participants are not representative of the entire population since the middle and upper income groups are under-represented. These are free health centers which have large patient populations and long waiting times and attract those of a lower socio-economic status. We also know nothing of the non-responders; their non-participation might have possibly skewed the results. A major weakness is that the instruments employed are screening and not confirmatory tests. Also because this was a cross-sectional study we can draw no conclusion on the causality of violence and mental illness so these results set the scene for further investigation using prospective studies. Future research should focus on refining the prevalence of the mental disorders found and confirming the findings using validated diagnostic tests. Additionally we need to document if the patient's quality of life is presently severely enough affected such that treatment was required and subsequently if this treatment was successful over time.

These results have a myriad of implications for primary care first contact clinics in Trinidad. Firstly these clinics and similar ones in Trinidad have a history of very large numbers of patients, and very short consultation times [22]. Yet results from studies such as this and those reported above [1-5] support the international findings [10,12,13,23] of a high level of mental health morbidity among primary care patients of woman abuse and other conditions including depression, anxiety, and posttraumatic stress disorder. Such conditions require a much longer consultation to recognize and initiate treatment plus sizable resources to support the primary care worker. It has been well documented that mental health continues to be under-funded, under-researched and poorly resourced in the developing world [24]. Papers such as this one continue to provide advocates with the evidence to entreat the political powers to provide the financial and human resources required to address these issues and educators with the conviction to train their students with these curricula.

## Conclusions

This cross-sectional survey of 432 women of primarily lower socioeconomic status who attend walk-in clinics in Trinidad revealed that abuse as measured by the Woman Abuse Screening Test (WAST)-Short scale is high. There were statistically significant associations between abuse and mental disorders such as depression, suicidal ideation, post-traumatic stress disorder and somatization, as determined by the Primary Care Evaluation of Mental Disorders (PRIME-MD) scale.

## Competing interests

The authors declare that they have no competing interests.

## Authors' contributions

All authors contributed to the conception and design; all authors except RGM assisted in the acquisition of data; all authors contributed to the analysis and interpretation of data and have been involved in the drafting of the manuscript or revising it critically for important intellectual content; and have given final approval of the version to be published.

## Pre-publication history

The pre-publication history for this paper can be accessed here:

http://www.biomedcentral.com/1471-2296/11/26/prepub
